# Comparison of RNA extraction kits and histological stains for laser capture microdissected prostate tissue

**DOI:** 10.1186/s13104-015-1813-5

**Published:** 2016-01-07

**Authors:** Kimberley Kolijn, Geert J. L. H. van Leenders

**Affiliations:** Department of Pathology, Erasmus MC, Rotterdam, The Netherlands

**Keywords:** Laser capture microdissection, RNA extraction kit, Prostate

## Abstract

**Background:**

Laser capture microdissection offers unique possibilities for the isolation of specific cell populations or histological structures. However, isolation of RNA from microdissected tissue is challenging due to degradation and minimal yield of RNA during laser capture microdissection (LCM). Our aim was to optimize the isolation of high-quality RNA from laser capture microdissected fresh frozen prostate tissue on the level of staining and RNA extraction.

**Results:**

Cresyl violet and haematoxylin were compared as histological stains for LCM. While RNA quality was similar for cresyl violet (median RIN 7.4) and haematoxylin (median RIN 7.6), tissue morphology was more detailed with cresyl violet as compared to haematoxylin. RNA quality from the following kits was compared: RNeasy^®^ Micro (median RIN 7.2), miRNeasy Mini (median RIN 6.6), Picopure^®^ (median RIN 6.0), mirVana™ miRNA (median RIN 6.5) and RNAqueous^®^-Micro (median RIN 6.3). RNA quality from microdissected samples with either the RNeasy Micro or miRNeasy Mini kit, was comparable to RNA isolated directly from whole tissue slices (median RIN 7.5, p = 0.09). Isolated RNA from benign and prostate cancer microdissected tissue demonstrated that RNA quality can vary between regions from the same clinical sample. Additionally, RNA quality (r = 0.89), but not quantity (r = 0.69) could be precisely measured with the Agilent Bioanalyzer.

**Conclusions:**

We demonstrate that staining with cresyl violet results in the isolation of high quality RNA from laser capture microdissected tissue with high discriminative morphology. The RNeasy Micro and miRNeasy Mini RNA extraction kits generated the highest quality RNA compared to Picopure, mirVana and RNAqueous with minimal loss of RNA quality during LCM.

**Electronic supplementary material:**

The online version of this article (doi:10.1186/s13104-015-1813-5) contains supplementary material, which is available to authorized users.

## Findings

Biological and histological heterogeneity of tissue samples are challenging issues for the isolation and characterization of individual cell populations that typically comprise tissues such as epithelial, mesenchymal and immune cells. Techniques such as macrodissection produce samples consisting of a mixture of cell types. Laser capture microdissection (LCM) is a method to obtain samples containing one specific cell type from complex tissues based on phenotypical, cytological and histopathological features. Generally, stained tissue slices are observed in an inverted light microscope and under visual guidance, areas of interest are cut loose using a focused laser beam. Excised fragments are catapulted into a specialized tube and protein, RNA or DNA are subsequently extracted for molecular profiling techniques such as gene-expression analysis or proteomics. The extraction of RNA from fresh frozen prostate tissue is particularly challenging due to its rapid degradation by RNases and low quantities of material usually obtained through LCM.

**Fig. 1:**
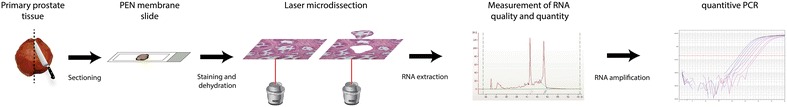
Procedural overview for the isolation of RNA from fresh frozen microdissected prostate tissue. Primary prostate tissue was sectioned and the sections were transferred to a membrane slide. After staining, areas of interest were microscopically identified and excised by microdissection. RNA was extracted from the tissue fragments using one of several commercial kits. Quality and quantity of RNA were measured with the Agilent Bioanalyzer. In a selection of RNA samples, the amount of two transcripts characteristic of prostate cancer were measured by quantitative PCR

In this study, we describe a protocol for the isolation of high quality RNA from fresh frozen prostate tissue including tissue handling, staining and RNA extraction kits of procured RNA (Fig. 1, Additional file 1).

**Fig. 2:**
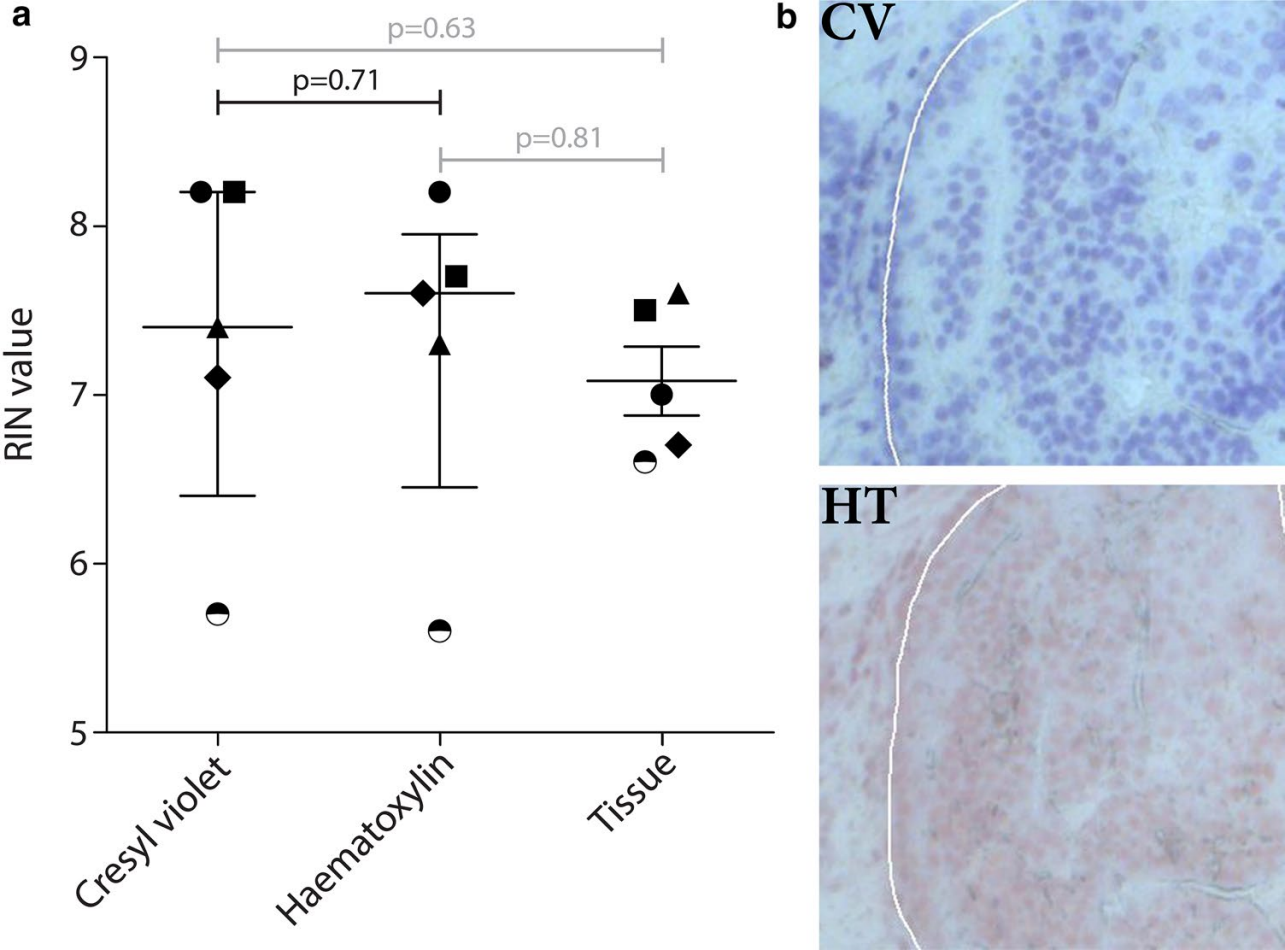
RNA quality of microdissected prostate cancer tissue stained with cresyl violet or haematoxylin. Consecutive slides from the same patient were stained with either cresyl violet (CV) or haematoxylin (HT), and matching tumor regions were isolated through LCM using whole tissue as control. While RIN values of microdissected tissues stained for cresyl violet or haematoxylin were similar (**a**), the morphology of cresyl violet stained slides was significantly more detailed than haematoxylin stained slides (**b**). Symbols represent material from the same patient. Median RIN values are shown, whiskers indicate interquartile range

To obtain high quality RNA from any type of tissue, optimization of steps that precede the LCM process are essential. In order to perform LCM on tissue slides, a histological stain is needed to discern the various cell types and structures present in the tissue. A widely used stain is haematoxylin, a staining method based on the reaction of haematein with DNA [1]. Metal ions in the DNA are used a mordant, giving a blue color to the nuclei. Cresyl violet or NissI staining uses a basic aniline dye that stains RNA, and thus the endoplasmic reticulum and ribosomes blue [2]. Others have compared these histological stains for other tissue types and cell lines, but not microdissected prostate cancer (PCa) tissue [3, 4]. We have compared the RNA quality of microdissected PCa tissue stained with haematoxylin or cresyl violet (Fig. 2a). Consecutive PCa tissue sections were stained with haematoxylin (n = 5) and cresyl violet (n = 5) and matching tumor regions from the same patient were isolated through LCM. As a control, RNA was isolated from one complete tissue slice immediately after cutting. The quality of RNA was subsequently measured with the Agilent Bioanalyzer as RNA integrity number (RIN).

The quality of RNA was similar for cresyl violet (median RIN 7.4) and haematoxylin (median RIN 7.6, p = 0.71) stained sections. However, the morphology of the tissue was much better visualized in cresyl violet treated slides (Fig. 2b). Studies have demonstrated that cresyl violet staining is also preferred for RNA isolation in breast and endometrium [5, 6]. Therefore, we applied cresyl violet staining for all further optimization steps.

### RNA quality is dependent on RNA extraction kit

There are several commercial RNA extraction kits available that allow the isolation of RNA from tissue such as RNeasy^®^ Micro (RNeasy), miRNeasy Mini (miRNeasy), Arcturus^®^ Picopure^®^ RNA isolation kit (Picopure), mirVana™ miRNA isolation kit (mirVana) and RNAqueous^®^-Micro (RNAqueous). Several studies have compared different RNA extraction kits on samples other than microdissected tissue [7–9]. Additionally, the preparation and process of LCM has been optimized for various tissues, including prostate [3, 10, 11]. However, to our knowledge there have been no reports on which RNA extraction kit is most suited for the isolation of RNA from microdissected (prostate) tissue. Therefore, we sought out to determine which RNA extraction kits can provide the best quality from fresh frozen prostate tissue fragments isolated through LCM.

RNA was extracted in parallel from microdissected PCa and benign regions from three patients in duplicate using each of the 5 RNA isolation kits (total of 60 LCM samples). Matching tissue regions (PCa and benign) were microdissected from the prostate tissue with a similar surface area (7 mm^2^). Additionally, RNA was isolated from a whole tissue slice (10 µm) from the same patient in duplicate with each kit, as a control for potential LCM quality loss (n = 30). PCa cell lines PC3, LNCaP and DU145 served as a control for high quality RNA control during each isolation round (n = 20) [12]. The duration of the LCM was kept within a maximum of 1 h to minimize RNA degradation, although RNA quality remained stable for at least 2 h (data not shown).

**Fig. 3:**
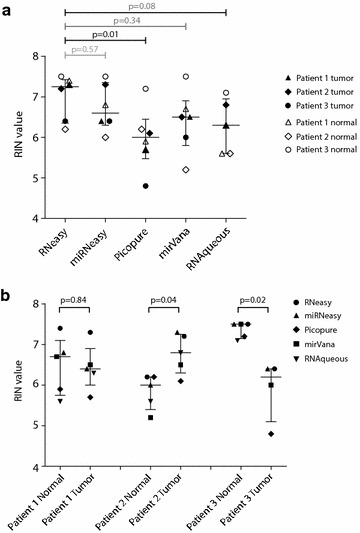
Comparison of RNA extraction kits for microdissected samples. RNA quality for benign and PCa LCM samples are shown for all RNA extraction kits. RIN values are shown as the average of a duplicate experiment. RNeasy and miRNeasy consistently delivered high quality RNA from LCM samples (**a**). Median RIN values were compared between the groups, which were expected to be similar. However, RNeasy generated higher quality RNA than Picopure and RNAqueous (p = 0.01 and 0.08 respectively). Additionally, RNA quality between benign glands and PCa cells within the same patient was variable (**b**)

First, we compared RIN values of RNA isolated with the RNeasy (median RIN 7.2), miRNeasy (median RIN 6.6), Picopure (median RIN 6.0), Mirvana (median RIN 6.5) and RNAqueous (median RIN 6.3) extraction kits, respectively (Fig. 3a). The main difference between the RNeasy and miRNeasy kits is the option in the latter to enrich for micro RNAs in the sample. The RNeasy and miRNeasy kits gave the best quality RNA and led to successful RNA isolation of every sample. Both the mirVana (2 out of 12 samples) and RNAqueous (3 out of 12 samples) kits failed more often to isolate RNA from microdissected tissue. RIN values of the RNeasy samples were significantly higher than those obtained with the Picopure (p = 0.01) samples, but the differences were not found to be significant with RNAqueous (p = 0.08), miRNeasy (p = 0.57) or mirVana (p = 0.34). However, the number of samples was too low to conclude that the RNeasy and miRNEasy kit are truly superior to the other tested RNA extraction kits. Additionally, we found that the quality of extracted RNA can vary between benign and PCa areas of the same slide (Fig. 3b). In the first patient, RNA extracted from benign tissue showed similar quality (median RIN 6.7) as that extracted from PCa tissue (median RIN 6.4, p = 0.84). However, in the second (RIN 6.0 vs 6.8) and third patient (RIN 7.5 vs 6.2), RNA isolated from benign tissue and PCa cells was significantly different (p = 0.04 and p = 0.02 respectively). This difference may be due to local changes in ribonuclease activity. Studies on the expression of RNases in tumors are contradictory, although it has been suggested that RNase activity decreases in rapidly growing tumors [13]. The amount of blood loss during prostatectomy and tumor stroma content have also been shown to affect RNA degradation [14]. However, RNA quality can also vary in general throughout tissue slices, independent of morphology.

**Fig. 4:**
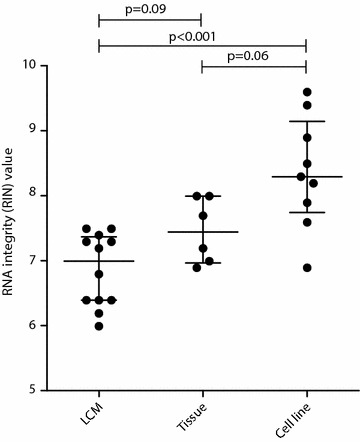
RNA integrity values for material isolated from LCM samples, whole tissue slices and cell lines. RNA quality (RIN values) for LCM derived samples, whole tissue slices and cell lines isolated with the RNeasy or miRNeasy kits. Median RIN values are shown, whiskers indicate interquartile range

The overall quality of RNA obtained with the RNeasy or miRNeasy kit from control cell lines (n = 9), whole tissue sections (n = 12) and benign and PCa LCM material (n = 24) was compared (Fig. 4). As expected, the average quality of RNA obtained from PCa cell lines was significantly higher (median RIN 8.3) than from LCM material (median RIN 7.0, p < 0.001). However, the LCM samples still generated high quality RNA that was comparable to RNA from whole tissue sections from the same patients (median RIN 7.5, p = 0.09). Therefore, we demonstrate that high quality RNA can be isolated from microdissected tissue with only a small quality loss (±0.5 RIN) compared to whole tissue slices. Additionally, we found that the RNA quality of whole tissue sections was a good indication on the RNA quality of microdissected material taken from the same tissue sections. We would therefore recommend to test overall tissue quality prior to LCM. To confirm that the extracted RNA was reflective of the morphological origin (benign versus PCa), we performed amplification and quantitative PCR on RNA from normal (n = 3) and PCa (n = 3) LCM (RNeasy) samples with primers directed against the metabolic enzyme alpha-methylacyl-coenzyme A racemace (AMACR) and the serine protease hepsin. These proteins are frequently overexpressed in PCa [15–17]. *AMACR* and *hepsin* were upregulated 40- and 13- fold respectively in all three tested PCa samples compared to the respective benign tissue (results not shown). Overall, these results demonstrates that RNA isolated from LCM samples with our workflow provides high quality RNA, which could be used for further downstream analyses. A complete step by step protocol for the isolation of high quality RNA from prostate tissue is included as a supplementary file (Additional file 1).

### RNA quality, not quantity, can be precisely measured with the Agilent Bioanalyzer

**Fig. 5:**
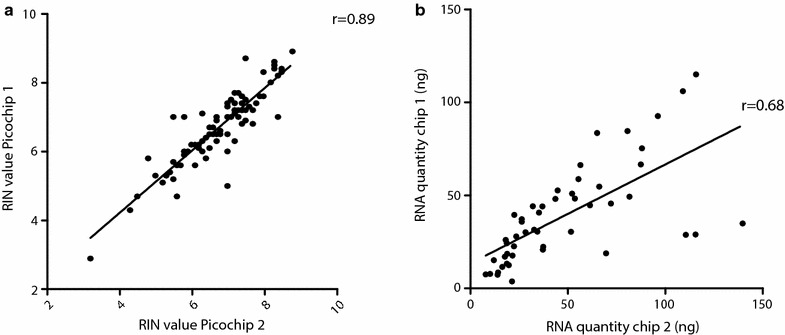
Correlation between RIN values and RNA quantities of samples between microfluidic chips. RNA quality (**a**) and quantity (**b**) for LCM samples, PCa cell lines and whole tissue sections was measured in two independent Agilent Bioanalyzer microfluidic chips. The correlation between measured RNA quality values was high (r = 0.89). Measurement of RNA quantity level varied between duplicate readings (r = 0.68)

The Bioanalyzer is a very sensitive instrument that can measure picograms of RNA material. However, this also implies that contaminants can have a major impact on the final measurements. For example, we often encountered ‘ghost peaks’ in the electropherograms generated by the Bioanalyzer that would impede the interpretation of the electropherogram. Therefore, we tested the reproducibility of RNA quality and quantity measurements with the Bioanalyzer by comparing the results obtained in duplicate analyses of the same samples (Fig. 5). RIN values measured in the same sample with different microfluidic chips were strongly correlated (Fig. 5a, r = 0.89). However, the correlation between RNA quantities was low (Fig. 5b, r = 0.68). As the quantities of RNA samples are based on the ladder of each individual microfluidic chip, variations between ladder batches may attribute to the low correlation between quantity measurements. In conclusion, RIN values are consistently measured with a Bioanalyzer, but not RNA quantity.

The isolation of high-quality RNA from LCM material is a major challenge. In this study, we provide a working protocol for the isolation of high quality RNA from fresh frozen prostate tissue (Additional file 1). We found that the use of cresyl violet as a histological stains permits the isolation of high quality RNA from LCM prostate tissue with good discriminative tissue morphology. We also demonstrated that the applied RNA extraction kit can influence the quality of isolated RNA and that the RNeasy and miRNeasy kits consistently deliver high quality samples reflective of morphological origin. Furthermore, the RNA quality can vary within the same tissue slice. Finally, we showed that the Agilent Bioanalyzer can reproducibly determine RNA quality, but not quantity. This should be taken into consideration when samples are subsequently used for experiments that require precise input quantities.

## Methods

### Clinical specimens and prostate cancer cell lines

Radical prostatectomy (RP) specimens were derived from the archive of the department of pathology, Erasmus Medical Centre, Rotterdam, The Netherlands. All RP’s had been performed in our institute between July 2010 and May 2013, prompted by histologically proven hormone-naïve PCa on diagnostic needle-biopsy. After receipt of RP specimens at the department of pathology, a transverse tissue slice was frozen in liquid nitrogen for research purposes and evaluated by a urogenital pathologist (GvL). Patients in Erasmus MC consent with the use of remaining tissue for research purposes based on an opt-out procedure, which was approved by the local Medical Ethical Committee (MEC 2011-195 and 2011-196). The opt-out procedure is a national well-accepted regulatory method during which the patient is informed verbally and can object against the use of the remaining tissue in written form. This procedure was developed by the FEDERA (Federation of Dutch Medical Scientific societies) and described in an official code of conduct (Human Tissue and Medical Research: Code of conduct for responsible use, 2011). Prostate cancer cells lines PC3, LNCaP and DU145 were used as a control for isolation of high quality RNA. Cell lines were obtained from the American Type Culture Collection (ATCC, Rockville, MD, USA) and maintained at 37 °C/5 % CO_2_ in RPMI 1640 containing 5 % fetal calf serum (Beohringer, Germany) and penicillin/streptomycin (P/S) (Lonza, Belgium).

### Fresh frozen tissue handling and laser capture microdissection

Fresh frozen prostate tissue was stored in liquid nitrogen or at -80 °C. Polyethylene naphthalate (PEN) membrane slides (MembraneSlide 1.0 PEN, Carl Zeiss, Germany) were pretreated under a UV light (UV Curing Lamp, Instrumedics Inc., USA.) for 30 min. Tissues from three patients were cut in 10 µm slices in a cryostat (Microm HM 560, Adamas Instrumenten, the Netherlands) at −26 °C with Tissue-Tek O.C.T. Compound (Sakura Finetek Europe, the Netherlands) and mounted on PEN slides. Slides were immediately transferred to dry ice in slide boxes (Sliderite 5 mailer, CellPath, U.K.), wrapped in tin foil and stored at −80 °C. One whole tissue slice was cut, transferred to a Eppendorf tube and stored −80 °C as control. For staining and dehydration of tissue, frozen slices were transferred directly to the following pre-cooled solutions on ice: 70 % ethanol for 1 min, distilled water (Gibco, Life Technologies, USA.) for 30 s, 1 % cresyl violet in 50 % ethanol (Cresyl Violet acetate, Sigma–Aldrich, the Netherlands) or haematoxylin (Klinipath, the Netherlands) for 2 min, 70 % ethanol for 1 min and 100 % ethanol for 1 min. Slides were immediately processed with LCM using a Zeiss PALM^®^ Microbeam IV (Carl Zeiss, Germany) at room temperature for a maximum of 1 h. Laser focus and energy settings were optimized for every slide to minimize damage from laser cutting of the tissue. Tissue was captured in AdhesiveCap500 opaque tubes (Carl Zeiss, Germany), put on dry ice and stored at −80 °C.

### RNA extraction and measurement

The following RNA extraction kits were used to isolate total RNA from LCM acquired material: RNeasy^®^ Micro (Qiagen, Germany), miRNeasy Mini (Qiagen, Germany), Arcturus^®^ Picopure^®^ RNA isolation kit (Arcturus, Applied Biosystems, USA.), mirVana™ miRNA isolation kit (Ambion, USA.) and RNAqueous^®^-Micro (Ambion, USA.). Samples were taken from a −80 °C freezer and lysis buffer from the according RNA extraction kit was immediately added to the sample at room temperature. Buffer and tissue were mixed for 30 s through pipetting and left on the bench for 5 min to assure proper lysis. Extraction was performed according to manufacturer’s protocols with an on-column DNAse digestion step (RNase-Free DNase set, Qiagen, Germany) added after the first wash buffer step for each kit. RNA quality and quantity was measured in duplicate with a 2100 Bioanalyzer and the RNA 6000 Pico Kit according to manufacturer’s protocol (Agilent Technologies, USA.). An additional water wash step of the Bionalyzer electrodes was added prior to PicoChip analysis to minimize RNaseZap (Applied Biosystems, USA.) associated ghost peaks.

### Statistics

Median RIN values from cresyl violet and haematoxylin LCM samples were compared with a Wilcoxon matched-pairs signed rank test. The non-parametric Mann–Whitney U test was used to compare median RIN values for RNA quality from LCM, cell lines and tissue samples from the observed differences between groups. Correlation of RIN values and RNA quantity between chips was calculated with Pearson’s r test. All graphs and statistics were performed in GraphPad Prism (version 3.0). p values <0.05 were considered significant.

## Availability of supporting data

The data sets supporting the results of this article are included within the article and its additional files.

## Additional file

10.1186/s13104-015-1813-5 Complete working protocol for RNA extraction from laser capture microdissected (LCM) tissue samples.

## Abbreviations

AMACR: alpha-methylacyl-coenzyme A racemace
LCM: laser capture microdissection
PCa: prostate cancer
RIN: RNA integrity number
RP: radical prostatectomy
