# Supplementary material for: Comparison of RNA extraction kits and histological stains for laser capture microdissected prostate tissue
Source: BMC Res Notes. 2016 Jan 7;9:17. doi: 10.1186/s13104-015-1813-5 (PMC4705748; doi:10.1186/s13104-015-1813-5)
Supplement: Supplementary file 1 — 10.1186/s13104-015-1813-5 Complete working protocol for RNA extraction from laser capture microdissected (LCM) tissue samples. [file 13104_2015_1813_MOESM1_ESM.docx]

Complete working protocol for RNA extraction from laser capture microdissected (LCM) tissue samples

**Tissue cutting**

- Store obtained tissue at -80°C to minimize RNA degradation.
- Irradiate membrane slides (MembraneSlide 1.0 PEN, Carl Zeiss, Germany) for 30 minutes under UV light before cutting (destroy bacteria and activate membrane)
- Clean all equipment/utensils (preferably also the knife) at room temperature with 70% ethanol followed by RNaseZap (Applied Biosystems, U.S.A.). Clean all other cold objects with 70% ethanol.
- Avoid defrosting of tissue, work as fast as possible. Cut tissue at the desired thickness (usually 10 µm) and transfer immediately to UV-treated membrane slide and into a slidebox on dry ice.
- Store slidebox at -80°C until further processing.

**Laser Capture Microdissection**

- Prepare solutions in clean 50 ml tubes with milliQ and 100% ethanol (preferably use an unopened bottle) and put on ice an hour in advance. Perform all staining steps on ice:
- Dehydrate in 70% ethanol for 1 minute
- Wash with milliQ for 30 seconds (to remove residual O.C.T. compound from cutting)
- Stain in 1% cresyl violet (Cresyl Violet acetate, Sigma Aldrich, the Netherlands) in 50% ethanol for 2 minutes
- Wash and dehydrate with 70% ethanol for 1 minute
- Wash and dehydrate with 100% ethanol for 1 minute

Ethanol steps are used to dehydrate tissue (e.g. displace water from tissue) and thus minimize RNase activity.

- Transfer slide(s) to clean slidebox for transport and perform LCM on tissue for 1 hour. We recommend to determine the appropriate LCM settings with dispensable tissue and the cut laser adjustment wizard

**RNA extraction**

- Perform RNA extraction (RNeasy Micro) according to manufacturer’s protocol and include the optional DNase digestion step.
- Perform RNA extraction rapidly, so avoid the extraction of more than 8 samples. Add a small amount (50-100µl) of lysis buffer to the tissue fragments in the AdhesiveCap (Carl Zeiss, Germany) lid. Pipet rapidly up and down for 30 seconds to lyse tissue thoroughly and transfer to a new tube containing the remaining lysis buffer.
